# Patterns of the Satisfaction and Frustration of Psychological Needs and Their Associations with Adolescent Students’ School Affect, Burnout, and Achievement

**DOI:** 10.3390/jintelligence11060111

**Published:** 2023-06-03

**Authors:** Linjia Zhang, Yi Jiang

**Affiliations:** Department of Educational Psychology, Faculty of Education, East China Normal University, Shanghai 200062, China

**Keywords:** self-determination theory, need satisfaction, need frustration, latent profile analysis, school functioning

## Abstract

Based on self-determination theory and adopting a person-oriented approach, we aimed to investigate the latent profiles of adolescent students’ basic psychological needs and their associations with personal characteristics (gender, socioeconomic status) as well as school functioning (school affect, burnout, academic achievement). Latent profile analyses based on a group of 1521 Chinese high school students identified four need profiles: *low satisfaction/moderate frustration*, *high satisfaction/low frustration*, *average all*, and *moderate satisfaction/high frustration*. Furthermore, there were significant differences in students’ school functioning among the four latent profiles. Specifically, students with moderate to high levels of need frustration were most likely to experience maladaptive school functioning, regardless of their need satisfaction level. Additionally, gender and socioeconomic status were significant predictors of profile membership. The findings of this study can assist educators in gaining a better understanding of the diverse patterns of psychological needs among students and help them to implement targeted interventions.

## 1. Introduction

Adolescent students differ greatly in their enthusiasm for school tasks. Some students engage in assigned tasks with enjoyment, while others are exhausted and stressed, putting minimal effort into school tasks. The reason behind such differences has been a focus of educational research. In the present study, we aimed to explore potential explanations for these differences based on a self-determination perspective (Deci and Ryan 2000; Ryan and Deci 2017).

As a macro-organism theory of motivational and personality development, self-determination theory (SDT) emphasizes the importance of basic psychological needs (i.e., the needs for autonomy, relatedness, and competence) for students’ academic functioning (Deci et al. 1991). Previous empirical research based on SDT has confirmed that need-satisfying experiences can catalyze high school and college students’ classroom engagement, personal growth, and well-being (e.g., Jang et al. 2016a, 2016b). Lately, scholars have shown a growing interest in need frustration as a way to comprehend the dynamics of basic psychological needs among adolescent students (Vansteenkiste et al. 2020). Existing research on this topic typically adopts a variable-oriented approach, which focuses on examining linear relationships among variables using statistical methods. However, this approach fails to capture the heterogeneity within the student population. In contrast, a person-oriented approach, such as latent profile analysis (LPA), examines how variables combine to form profiles and how these patterns relate differentially to predictors and outcomes. Despite the considerable advantages of this approach, LPA remains relatively underutilized in this field.

To date, only a handful of studies have delved into the intraindividual patterns of basic psychological needs satisfaction and frustration within the framework of SDT, and this line of research can be further improved (C. Li et al. 2021; R. Li et al. 2020; Rouse et al. 2020; Warburton et al. 2020). For instance, need profiles of high school students within the educational context remain largely underexplored. High school education serves as a critical link leading to higher education. To optimize high school students’ learning process, it is necessary to investigate the characteristics of need profiles that emerge among students. In addition, limited evidence is available regarding how need profiles may be related to students’ demographic characteristics and school functioning. Furthermore, most existing studies on the topic of need profiles are based on participants from Western cultures; hence, more studies are needed to explore the patterns of such profiles in non-Western cultures. In the present study, we aimed to fill these gaps by using LPA to explore the combined patterns of three basic psychological needs—both their satisfaction and frustration—among Chinese high school students. We also examined whether and how distinct profiles may relate to school affect, burnout, and academic achievement. Furthermore, we investigated how students’ personal characteristics, namely, gender and socioeconomic status (SES), predict their need profile membership.

### 1.1. Basic Psychological Needs and School Functioning

According to SDT, human beings primarily have three basic psychological needs: the needs for autonomy, relatedness, and competence. These needs are innate, intrinsic, and considered universal across cultures (Chen et al. 2015; Deci and Ryan 2000). It is important to note that these needs can be either satisfied or frustrated. Specifically, autonomy satisfaction refers to a sense of choice and volition in one’s actions, whereas autonomy frustration refers to the experience of being pressured or pushed in an unwanted direction. Relatedness satisfaction pertains to a sense of belongingness and authentic connections with others, while relatedness frustration denotes a feeling of social estrangement, exclusion, and solitude. Competence satisfaction encompasses a perception of being capable and proficient in accomplishing one’s objectives, whereas competence frustration entails sentiments of inadequacy and uncertainty regarding one’s efficacy (Chen et al. 2015; Vansteenkiste and Ryan 2013).

Basic psychological needs are essential nutrients, the satisfaction and frustration of which can substantially account for both the “dark” and “bright” side of individuals’ functioning (Vansteenkiste et al. 2020). The satisfaction of these needs fosters individuals’ psychological growth, wellness, and well-being. On the other hand, the frustration of basic needs directly leads to depression, maladjustment, and ill-being (Vansteenkiste et al. 2020). Within the education context, the satisfaction of basic psychological needs is believed to have a crucial impact on students’ intrinsic motivation and optimal learning outcomes. Conversely, when these needs are frustrated, students tend to exhibit poor academic performance and maladaptive functioning. Thus, basic psychological needs are considered one of the important factors affecting students’ school functioning.

Among the various educational outcomes, school affect is one of the key indicators that measures students’ well-being at school. School affect includes both a positive aspect (e.g., relaxation, pleasure, and happiness) and negative aspect (e.g., depression, distress, and boredom; Pekrun et al. 2002; Tian et al. 2016). Adolescent students usually report a decreased positive affect at school as they progress to higher grades (Wigfield et al. 2015). In addition, school burnout occurs when students continually have difficulty coping with academic stress. School burnout specifically refers to a state of exhaustion in which students feel tired and stressed about school demands or schoolwork (Salmela-Aro et al. 2009). Previous studies have found that need satisfaction and frustration have a significant impact on students’ school affect, school burnout, and academic achievement. For example, in a study conducted by Vandenkerckhove et al. (2019), it was found that positive school affect showed a positive correlation with weekly variations in need satisfaction, whereas negative school affect exhibited a positive correlation with weekly variations in need frustration. Similarly, Tian et al. (2016) found that Chinese adolescents who experienced higher levels of need satisfaction in school reported more positive school affect and lower levels of negative school affect. Jang et al. (2012) also identified a negative relationship between the satisfaction of basic psychological needs and school burnout among Korean middle school students. Similar findings were observed in studies involving Chinese elementary and middle school students (Luo et al. 2014; Zhang and Gao 2019). Additionally, Jang et al. (2009, 2012) discovered that need satisfaction experiences were associated with higher levels of student achievement. A cross-cultural study, which included data from China, also found that students with higher levels of need satisfaction were more likely to achieve better academic outcomes (Nalipay et al. 2020).

Although the existing literature is enlightening, it has largely been based on a set of variable-oriented approaches, assuming a homogeneity across sample individuals and concentrating on the linear relationship between variables. To date, very few SDT studies have used a person-oriented approach to explore whether and how the satisfaction and frustration of the psychological needs for autonomy, relatedness, and competence cohere and function together within the learning context. Vansteenkiste et al. (2020) noted that an avenue for future research was to move towards a person-oriented perspective to shed light on individuals’ need profiles. Hence, we explored this possibility and heeded the recommendation by Vansteenkiste et al. (2020) in the current study.

### 1.2. Profiles of Basic Psychological Needs

Adolescent students show considerable variation over time in their experiences of need satisfaction and need frustration (Ratelle and Duchesne 2014). As a complementary method to the variable-oriented approach, a person-oriented approach (e.g., LPA) allows researchers to gain a deeper insight into within-person combinations of need satisfaction and frustration rather than “slicing” an individual into different need-relevant dimensions (Vansteenkiste et al. 2020). Such combinations carry practical implications because educators can know which need profile a student falls into, allowing for more specific, tailored interventions for one or more basic needs.

To our knowledge, only a few studies have so far relied on a person-oriented approach to individuals’ need profiles. All these studies have focused on the measures of basic psychological need satisfaction and frustration. However, they have mainly focused on the fields of physical education (PE) and the work context. Specifically, using cluster analysis, Warburton et al. (2020) identified five profiles among high school PE students and athletes: *low satisfaction/high frustration*, *high satisfaction/low frustration*, *moderate satisfaction/moderate frustration*, *moderate satisfaction/low frustration*, and *moderate satisfaction/high frustration*. Likewise, C. Li et al. (2021) conducted a study on primary and secondary school students in Singapore, investigating need satisfaction and frustration concerning physical activity participation. The researchers utilized an LPA to classify the participants into four distinct profiles: *average*, *low satisfaction/above average frustration*, *very high satisfaction/very low frustration*, and *high satisfaction/very high frustration*. In the workforce, Rouse et al. (2020) conducted an LPA and identified five profiles among firefighters: *high satisfaction/low frustration*, *low satisfaction/very high frustration*, *very high competence satisfaction/high frustration*, *slightly above average satisfaction/slightly below average frustration*, and *slightly below average satisfaction/high frustration*. Additionally, in a study focusing on Chinese college students, R. Li et al. (2020) conducted an LPA and identified four profiles based on measures of need satisfaction and frustration in the context of social networking sites: *unsatisfied/frustrated*, *satisfied/un-frustrated*, *average*, and *satisfied/frustrated*. 

Although these findings can shed light on unique patterns of need satisfaction and frustration, they provide limited information. It is still difficult to determine how high school students’ combined experiences of need satisfaction and frustration may manifest in school life. Therefore, more work is needed to explore basic psychological need profiles among high school students within the learning setting.

### 1.3. Profile Differences in Personal Characteristics and Outcomes

Person-oriented methods not only allow for the identification of profiles but also pro-vide insights into how these profiles vary based on personal characteristics and their as-sociations with important outcomes. Understanding which individuals are more likely to be categorized into specific profiles deepens our understanding of these profiles. However, there has been limited research on the underrepresentation or overrepresentation of particular groups in different need profiles. To the best of our knowledge, only Warburton and colleagues (2020) have examined gender differences in need profile membership among high school students in England. Their findings indicated that the high satisfaction/low frustration profile had a higher proportion of males compared to the low satisfaction/high frustration and moderate satisfaction/moderate frustration profiles. Given the existing evidence of the association between need profiles and gender, we aimed to investigate whether gender would predict the membership of non-Western high school students in different profiles. In addition to gender, family socioeconomic status (SES) is a crucial determinant of health and well-being among adolescents (Currie et al. 2012; Di Domenico and Fournier 2014). Moreover, SES has been found to be linked to students’ basic psychological needs, with a high SES being associated with a greater need fulfillment and a low SES being linked to need frustration (Di Domenico and Fournier 2014; González et al. 2016). However, there is a lack of research on profile differentiation based on students’ SES. Therefore, further research is needed to explore this important issue.

Moreover, person-oriented studies have identified distinct need profiles, allowing for an examination of how profile membership is linked to a variety of outcomes. There are some notable differences in academic functioning among configurations of need satisfaction and frustration. For instance, Warburton et al. (2020) found that participants belonging to the *high satisfaction/low frustration* profile reported higher levels of intrinsic motivation, enjoyment, and well-being than those belonging to the *low satisfaction/high frustration* profile. In contrast, participants in the *low satisfaction/high frustration* profile reported higher levels of amotivation, external regulation, and burnout than those in the *high satisfaction/low frustration* profile. Similarly, R. Li et al. (2020) found that students in the *unsatisfied/frustrated* profile exhibited a higher risk of social media addiction than those in the *satisfied/un-frustrated* profile. In general, existing empirical evidence suggests that a combination of high need satisfaction and low need frustration yields the most adaptive outcomes (C. Li et al. 2021; R. Li et al. 2020; Rouse et al. 2020; Warburton et al. 2020). However, patterns of combined rather than separate effects of need satisfaction and frustration on important educational outcomes such as students’ school affect, burnout, and achievement remain underexplored. Thus, additional research on the topic is warranted.

### 1.4. The Present Study

Previous person-oriented research adopting the SDT framework has yielded some interesting findings. However, existing studies can be further expanded upon. The current study adopted an LPA to explore how subpopulations of high school students were characterized by a similar configuration of need satisfaction and frustration within the educational context. We also compared the resulting profiles with regard to students’ demographic characteristics, namely, gender and SES, and school functioning, namely, school affect, burnout, and achievement.

Based on the existing literature, we hypothesized there were at least two need profiles in high school settings. However, due to limited prior knowledge on need profiles within the high school context, we did not establish explicit hypotheses regarding the number and patterns of the latent profiles. Furthermore, drawing from previous studies and SDT propositions (Vansteenkiste et al. 2020; Warburton et al. 2020), we expected to identify distinct latent patterns that would vary in terms of students’ personal characteristics and school functioning.

## 2. Method

### 2.1. Participants 

Data for this study were collected from a regular academic-track public school in a metropolitan city in China. The school included junior and senior high school divisions covering grades 7 to 12. The sample consisted of 1521 grade 11 students, 45% being boys. The students were nested within 30 classes. The average age of the participants was 17.38 years (SD = 0.58). The survey was administered by teachers during regular school hours, and the students completed it online using computers. There were no missing data in the students’ survey responses. The study received approval from the institutional review board for human participants at the author’s university, and informed consent was obtained from all participating students. All procedures followed relevant institutional guidelines for the protection of human subjects and adhered to the ethical guidelines of the American Psychological Association (APA).

### 2.2. Measures

All survey items were written in Chinese and used six-point Likert-type scales ranging from 1 (*completely disagree*) to 6 (*completely agree*). To assess the reliability of the measures, Cronbach’s α coefficients were utilized. Scales that were initially developed in English were subjected to the translation and backtranslation process proposed by Brislin (1970). The survey items are included in Appendix A.

#### 2.2.1. Basic Psychological Need Satisfaction and Frustration

Students’ basic psychological needs were measured with the Chinese version of the Basic Psychological Need Satisfaction and Frustration Scale (BPNSNF) (Chen et al. 2015). The scale has 24 items that cover 6 dimensions, including autonomy satisfaction (e.g., “I feel that my decisions reflect what I really want”; *α* = 0.62), autonomy frustration (e.g., “I feel forced to do many things I wouldn’t choose to do”; *α* = 0.76), relatedness satisfaction (e.g., “I feel that the people I care about also care about me”; *α* = 0.76), relatedness frustration (e.g., “I feel excluded from the group I want to belong to”; *α* = 0.77), competence satisfaction (e.g., “I feel confident that I can do things well”; *α* = 0.82), competence frustration (e.g., “I feel disappointed with many of my performances”; *α* = 0.78).

#### 2.2.2. School Affect

Seven items measuring school affect from Kaplan and Maehr (1999) were adopted. There were three items for positive affect (e.g., “Most of the time, being in school puts me in a good mood”) and four items for negative affect (e.g., “School often makes me feel bad”). In the present study, the reliability coefficients of this scale were *α* = 0.84 for positive affect and *α* = 0.85 for negative affect.

#### 2.2.3. School Burnout

Four items measuring school burnout from the School Burnout Inventory (Salmela-Aro et al. 2009) were adopted. The School Burnout Inventory measures three dimensions of school burnout including exhaustion at schoolwork, cynicism toward the meaning of school, and sense of inadequacy at school, which are all separate constructs. For the research questions of interest in the present study, we used the exhaustion at schoolwork subscale to capture students’ maladaptive functioning in school learning settings. A sample item was “I feel overwhelmed by my schoolwork”. In the present study, the reliability coefficient for this subscale was *α* = 0.80. 

#### 2.2.4. Academic Achievement

Students’ scores on their end-of-semester final examination were provided by the school and used as an academic achievement index. The examination took place four weeks after the questionnaire and covered multiple school subjects, including Chinese, mathematics, English, physics, biology, politics, history, and geography. All students took the same exam and were scored in the same way using a standard answer key. The maximum possible score on this examination was 750. Students’ actual scores ranged from 136 to 654.

#### 2.2.5. Demographic Variables

The students reported their demographic information, which included their gender and SES. To measure the SES, we utilized eleven items from the Organization for Economic Cooperation and Development (OECD 2012). We computed the SES index based on total scores regarding parental education level (one item each for paternal and maternal education level, e.g., “What is the highest level of education your father has completed?” The four answer options for this question are: 1. secondary degree or below; 2. vocational college degree; 3. bachelor’s degree; 4. master’s degree or above), home possessions (seven items, e.g., “How many mobile phones do you have?” The four answer options for this question are: 1. 0; 2. 1; 3. 2; 4. 3 or above), and home educational resources (two items; e.g., “How many books do you have at home?” The five answer options for this question are: 1. 0–10; 2. 11–25; 3. 26–100; 4. 101–200; 5. 200 or above). This scale has been used effectively in prior research with Chinese adolescent students (e.g., Jiang and Zhang 2023; Wu et al. 2022). In the present study, SES scores ranged from 12 to 44 (*M* = 24.41, *SD* = 4.07), with higher scores indicating greater levels of SES.

### 2.3. Statistical Analysis 

The data were analyzed using Mplus 8.3 with a robust maximum likelihood estimator (MLR). To account for the nonindependence of data arising from the nesting of students within classes (McNeish et al. 2017), a design-based correction of standard errors was applied with Type = Complex. The missing data in achievement were addressed through the utilization of a full information maximum likelihood (FIML) estimation (Schafer and Graham 2002).

To identify the number of profiles that best reflected students’ need satisfaction and frustration, we used an LPA to examine models with two to eight profiles (k = 2–8). All LPA models were freely estimated with 1000 random starts, 100 iterations in the initial stage, and 200 final-stage optimizations. Our decision regarding the optimal number of profiles was based on both statistical evaluations and the conceptual grounding and interpretability of the profiles. We assessed the fit of the models using several fit indices, including the Akaike information criteria (AIC), Bayesian information criteria (BIC), sample-size-adjusted BIC (aBIC), and the Vuong–Lo–Mendell–Rubin likelihood ratio test (VLMR). Lower values of AIC, BIC, and aBIC suggest a better fit, while a significant *p* value of VLMR implies that the k-profile model fits better than the k-1 profile model. BIC, which has a high reliability for model fitting according to Nylund et al. (2007), was considered the primary reference indicator. Additionally, we inspected the entropy value, which ranges from 0 to 1 and describes the classification’s precision. A value above 0.70 indicates a relatively accurate classification.

Once the latent profile solution was identified, students were assigned to profiles based on their highest membership probability. To facilitate the interpretation of the profiles, z-scores were computed for each of the six profile indicators. In line with the criteria used in prior person-centered research (Rouse et al. 2020; Warburton et al. 2020), we evaluated whether a profile scored relatively “high”, “moderate”, or “low” on the profile indicators. Specifically, scores greater than or equal to ±1 SD were classified as very high/low, scores ranging from ±0.5 to 1 SD were classified as high/low, and scores ranging from −0.5 to 0.5 SD were classified as moderate.

To explore whether individual characteristics (i.e., gender and SES) predicted membership in the latent profiles, we utilized the R3STEP command for auxiliary variables available in Mplus 8.3 (Asparouhov and Muthén 2014a). The R3STEP approach automatically computes odds ratios (OR) and compares the likelihood of individuals being classified into one profile versus another, based on the covariate of interest (i.e., gender and SES). To investigate significant differences in the outcome variables (i.e., school affect, burnout, and achievement) across students’ need profiles, we used the automatic Bolck–Croon–Hagenaars (BCH) method recommended for an LPA with the outcome variables of interest in Mplus (Asparouhov and Muthén 2014b). This approach entails performing a weighted analysis of variance with the posterior profile membership probabilities as weights. Wald chi-square tests were used to assess differences in profile-specific means (Bakk and Vermunt 2016).

## 3. Results

### 3.1. Descriptive Statistics

The means, standard deviations, Cronbach’s alpha coefficients, and bivariate correlations for all variables are presented in Table 1. The correlations of the satisfaction and frustration of autonomy, relatedness, and competence with other variables were consistent with what has been shown in the existing literature. Specifically, the satisfaction of autonomy, relatedness, and competence correlated positively with positive affect (0.24 ≤ *rs* ≤ 0.32, *ps* < 0.001) and correlated negatively with negative affect (−0.33 ≤ *rs* ≤ −0.27, *ps* < 0.001) and school burnout (−0.24 ≤ *rs* ≤ −0.20, *ps* < 0.001). Competence satisfaction correlated positively with achievement (*r* = 0.09, *p* < 0.001). The frustration of autonomy, relatedness, and competence correlated positively with both negative affect (0.46 ≤ *rs* ≤ 0.56, *ps* < 0.001) and school burnout (0.40 ≤ *rs* ≤ 0.51, *ps* < 0.001), but correlated negatively with positive affect (−0.34 ≤ *rs* ≤ −0.23, *ps* < 0.001).

### 3.2. Identifying Profiles for Students’ Psychological Need Satisfaction and Frustration

Table 2 shows the fit criteria for the LPA models that were applied to the basic psychological need satisfaction and frustration data, with two to eight latent profiles being examined. In selecting the final model, we considered several factors, including conceptual grounding, parsimony, and profile interpretability. While AIC, BIC, and aBIC values decreased as the number of profiles increased, the BIC score leveled off from the four-profile to the five-profile solution, indicating that the latter did not offer any significant improvement. Furthermore, the profiles in the five-profile solution showed some degree of overlap. We also took into account prior research and the theoretical underpinnings of basic psychological need theory, which suggested that our sample was unlikely to be neatly divided into two groups. After evaluating the interpretability of the three- and four-profile solutions, we determined that the latter was more theoretically sound and consistent with prior research. Although some students in the four-profile solution were not a perfect match for any one of the groups identified in the three-profile solution, this model had a higher entropy value of 0.75, indicating a good classification precision. In addition, Table 3 provides the probability scores that support a clear classification of students into their most likely latent profiles. Based on these considerations, we ultimately selected the four-profile solution as our final model.

**Figure 1:**
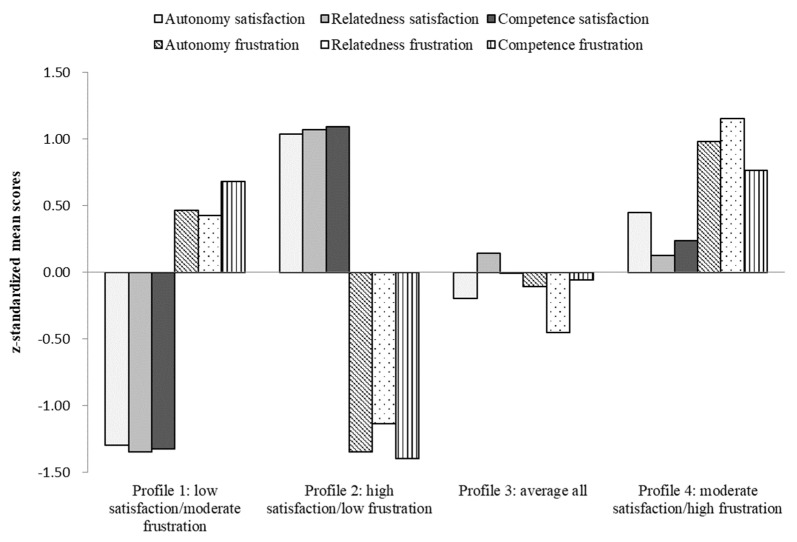
Latent profiles with z-standardized mean scores of indicators.

The four distinct profiles were labelled based on their mean values of basic psychological need satisfaction and frustration (see Table 4 and Figure 1). The first profile (*N* = 316; 21% of the sample) was *low satisfaction/moderate frustration*, in which students reported very low need satisfaction along with moderate levels of need frustration. The second profile (*N* = 356; 23% of the sample) was *high satisfaction/low frustration*, in which students reported high satisfaction and very low frustration. The third profile was the largest (*N* = 777; 51% of the sample). Students in this profile reported moderate levels of both need satisfaction and frustration. We thus labeled this profile *average all.* The fourth profile was the smallest (*N* = 72; 5% of the sample) and was characterized by moderate levels of satisfaction and high frustration of all three needs. We labelled this profile *moderate satisfaction/high frustration*.

### 3.3. Personal Characteristics as Predictors of Profile Membership

We explored whether the demographic variables of gender and SES were associated with the students’ profile membership. As shown in Table 5, gender emerged as a significant predictor of students’ profile membership. Specifically, we found that female students were less likely to be assigned to the *moderate satisfaction/high frustration* profile than the *low satisfaction/moderate frustration* profile (OR = 0.46, *p* < 0.01), the *high satisfaction/low frustration* profile (OR = 0.57, *p* < 0.05), or the *all-average* profile (OR = 0.49, *p* < 0.01).

The students’ SES also emerged as a significant predictor of profile membership. Specifically, students with a higher SES were more likely to be assigned to the *high satisfaction/low frustration* than the *low satisfaction/moderate frustration* profile (OR = 1.06, *p* < 0.05). Students with a higher SES were more likely to be assigned to the *moderate satisfaction/high frustration* than the *low satisfaction/moderate frustration* profile (OR = 1.11, *p* < 0.05) or the *all*-*average* profile (OR = 1.10, *p* < 0.01). In addition, students with a higher SES were less likely to be assigned to the *all*-*average* than the *high satisfaction/low frustration* profile (OR = 0.95, *p* < 0.05).

### 3.4. Profile Differences Regarding School Affect, School Burnout, and Achievement

BCH analyses indicated that the four latent profiles significantly differed in their levels of the outcome variables apart from achievement (see Table 6). Mean comparisons further revealed differences in school affect and burnout between the different profiles. For a positive school affect, students in the *high satisfaction/low frustration* profile exhibited the highest scores, which were significantly higher than those of students in the *all-average*, *moderate satisfaction/high frustration*, and *low satisfaction/moderate frustration* profiles, sequentially (overall Wald *χ*^2^ = 36.38–244.33, *p* < 0.001). Students in the *all-average* profile had a significantly higher positive affect than students in the *low satisfaction/moderate frustration* profile (Wald *χ*^2^ = 40.35, *p* < 0.001). There were no statistically significant differences between the *all-average* and *moderate satisfaction/high frustration* profiles (Wald *χ*^2^ = 2.77, *p* = 0.10). Students in the *low satisfaction/moderate frustration* profile had the lowest scores. There were no statistically significant differences between the *low satisfaction/moderate frustration* and *moderate satisfaction/high frustration* profiles (Wald *χ*^2^ = 0.72, *p* = 0.40).

For negative school affect and burnout, students in the *moderate satisfaction/high frustration* and *low satisfaction/moderate frustration* profiles exhibited the highest scores, with no statistically significant differences between them (overall Wald *χ*^2^ = 3.27–3.40, *ps* = 0.07). Students in the *high satisfaction/low frustration* profile had the lowest scores, which were significantly lower than those of students in the *all-average*, *low satisfaction/moderate frustration*, and *moderate satisfaction/high frustration* profiles, sequentially (overall Wald *χ*^2^ = 12.82–191.87, *ps* < 0.001).

## 4. Discussion

Guided by SDT and a person-oriented perspective, the purpose of the present study was to examine which profile patterns of need satisfaction and frustration exist among high school students and how sociodemographic antecedents and school outcomes are linked with profile membership. Using an LPA, our findings revealed four distinct profiles representing different combinations of need satisfaction and frustration. Students’ gender and SES differentially predicted the need profile membership. Furthermore, significant differences in students’ school affect and burnout were observed across the different profiles.

### 4.1. Types and Characteristics of Need Profiles

In the present study, our results revealed four distinct profiles of needs satisfaction and frustration among Chinese high school students: (1) *low satisfaction/moderate frustration*, (2) *high satisfaction/low frustration*, (3) *average all*, and (4) *moderate satisfaction/high frustration*. Representing most of the sample, the *high satisfaction/low frustration* and *average all* profiles were consistent with combinations observed in previous person-oriented studies (C. Li et al. 2021; R. Li et al. 2020; Rouse et al. 2020; Warburton et al. 2020). This indicates that most students have an adaptive pattern of basic psychological needs, which is encouraging. Furthermore, these two types of profiles seem to exist among individuals across multiple contexts (e.g., PE, work, and social networking; C. Li et al. 2021; R. Li et al. 2020; Rouse et al. 2020; Warburton et al. 2020).

Although relatively smaller in size, the emergence of the *low satisfaction/moderate frustration* and *moderate satisfaction/high frustration* profiles is also worth noting. Students in these two profiles experienced very low to somewhat moderate levels of need satisfaction and moderate to high levels of need frustration. One potential reason for the emergence of these two profiles may be that the Chinese high school environment is quite demanding and competitive. In such an environment, Chinese high school students are likely to focus on extrinsic goals (e.g., pursing higher scores, external rewards, or social recognition). According to goal content theory (GCT), a subtheory of SDT, students are likely to experience less satisfaction of basic psychological needs when focusing on extrinsic goals. Research on GCT consistently upholds the notion that not all goals have an equal impact on well-being. Specifically, placing excessive emphasis on extrinsic goals can lead to external pressure and a reduction in the satisfaction of needs and overall well-being (Ryan and Deci 2019). In addition, in China’s high school system, there is a strong emphasis on preparing students for the National College Entrance Examination (also known as Gaokao). Accordingly, Chinese high school students are under a lot of pressure. In particular, students may perceive a lack of autonomous choice regarding their learning activities within the school context. Therefore, students may perceive moderate or even high levels of need frustration in this relatively controlling educational setting. In addition, the *moderate satisfaction/high frustration* profiles endorse the notion that high school students can experience both need frustration and satisfaction simultaneously (C. Li et al. 2021; R. Li et al. 2020; Warburton et al. 2020).

### 4.2. Predictors and Outcomes of Need Profile Membership

Beyond identifying need profiles, we were also interested in whether students’ demographic characteristics (i.e., gender and SES) were associated with their likelihood of membership in a particular need profile, along with the relationship between need profiles and students’ school functioning, including school affect, burnout, and academic achievement.

Our results showed that both gender and SES were significant predictors of profile membership. Regarding gender, girls had a lower likelihood of membership in the *moderate satisfaction/high frustration* profile compared to the other three types of profiles. This suggests that girls are less prone to experiencing high levels of need frustration. Previous research has found that girls are more sensitive than boys to satisfaction and frustration of their need for recognition (e.g., Rodríguez-Meirinhos et al. 2020). However, our findings suggest that girls are less likely than boys to perceive high levels of need frustration. One potential explanation for this pattern is that girls are less externally controlled than boys regarding their academic actions especially in traditional teacher-directed learning contexts (Schweder and Raufelder 2021; Vallerand et al. 1997). According to SDT, the amount of satisfaction or frustration with basic psychological demands is inextricably related with the process of internalization (Ryan and Deci 2019). In light of this, it is possible that higher levels of internalization pertaining to values and regulations among girls could potentially make their needs less vulnerable to frustration. Our findings also support the idea that boys may be more vulnerable to teacher neglect and rejection than girls, as previously reported by Opdenakker (2021). However, further research is needed to confirm this finding.

Regarding SES, students with a higher SES had a higher likelihood of membership in the most adaptive profile, *high satisfaction/low frustration*, than in the maladaptive profile, *low satisfaction/moderate frustration*. One possible explanation for this finding is that the SES may shape access to resources (e.g., Tobin et al. 2021). Individuals in high-status positions could have more access to resources than those in disadvantaged positions. Moreover, researchers have argued that feelings of need satisfaction could be differentially distributed across SES levels as opportunity, power, and social relation resources are similarly distributed (e.g., Turner et al. 1999). Another potential explanation is that a higher SES has been frequently linked to higher levels of parental engagement in learning activities with adolescents (Ren et al. 2021). Parental engagement may benefit students’ psychological need satisfaction, making it more likely for students to have an optimal need profile. However, we also found that higher-SES students had a higher likelihood of being in the *moderate satisfaction/high frustration* profile compared to the *all-average* and *low satisfaction/moderate frustration* profiles. This may be because inappropriate parental involvement, such as the use of psychological control and conditional rewards, has the potential to increase stress and lead to a frustration of the needs. It is worthwhile for future research to scrutinize the exact reasons for the emergence of these SES-related findings.

With regard to the associations between profile membership and school functioning, our results showed that profiles of need satisfaction and frustration are useful for understanding students’ school affect and school burnout, which are important for their academic thriving. The *high satisfaction/low frustration* profile displayed the most adaptive pattern of school functioning, as students in this profile reported higher levels of positive school affect as well as lower levels of negative school affect and school burnout. The finding is in line with previous research using the person-oriented approach, which found that individuals in the *high satisfaction/low frustration* profile had the highest level of well-being and enjoyment (C. Li et al. 2021; R. Li et al. 2020; Rouse et al. 2020; Warburton et al. 2020). It also supports SDT’s postulate that the satisfaction of basic needs is associated with the “bright” side and contributes to optimal functioning, while need frustration is associated with the “dark” side and leaves one prone to malfunctioning (Cheon et al. 2019; Ebersold et al. 2019; Vansteenkiste et al. 2020). In contrast, the *moderate satisfaction/high frustration* and *low satisfaction/moderate frustration* profiles were more closely related to maladaptive school functioning, including the highest levels of negative school affect and school burnout. Moreover, we found no significant difference between these two profiles regarding school affect or burnout. Previous studies have suggested that need satisfaction has a protective effect on individuals’ functioning (C. Li et al. 2021; Warburton et al. 2020). However, findings from the current study suggest that the possibly protective mechanism of need satisfaction may not function well at relatively low levels. In terms of academic achievement, we did not find any significant differences across the identified profiles. In the present study, students’ scores on end-of-semester final examinations were used as an indicator of academic achievement. This score integrates the outcomes of diverse subjects studied by the students in school and thus, to some extent, represents their comprehensive performance level. This implies that even high-achieving students may be classified into suboptimal profiles in terms of their psychological needs. It highlights the relevance of considering the distinct profiles of need satisfaction and frustration for students with varying academic performance levels. These profiles should be taken into careful consideration when addressing the well-being and educational experiences of students.

### 4.3. Implications

The present study’s findings have several theoretical and practical implications. First, we found that Chinese adolescent students differentiate between basic psychological need satisfaction and frustration. The emergence of the *moderate satisfaction/high frustration* and *low satisfaction/moderate frustration* profiles, which were not solely characterized by opposite experiences of need satisfaction and need frustration, suggests that students reported simultaneously experiencing both need frustration and need satisfaction in real learning contexts. These findings highlight the necessity of considering both the “bright” and “dark” sides of student basic psychological needs, a perspective that has gained increased attention in recent years (Vansteenkiste et al. 2020). Furthermore, the present results also reveal a meaningful combined effect of need satisfaction and need frustration on students’ academic outcomes. In particular, need frustration may undermine the protective impact of need satisfaction, leading to maladjustment. Therefore, it would be beneficial to treat need frustration as an independent construct to prevent it from exerting its negative potential influence on students’ psychological functioning.

In terms of practical implication, our results provide useful information for tailoring interventions based on the specific pattern of students’ need satisfaction and frustration. For example, the identified need profiles allow instructors to target maladaptive subgroups of high school students, such as the *moderate satisfaction/high frustration* and *low satisfaction/moderate frustration* profiles. Students in these profiles experienced either very high levels of need frustration or very low levels of need satisfaction. They also displayed more maladjusted school functioning than students in other profiles, such as high levels of negative school affect and school burnout. Therefore, these students need more care and guidance from instructors. In addition, instructors should pay attention to the differences in learning outcomes among students with distinct need profiles. Although we did not find significant differences in academic achievement across profiles, students’ affective-related outcomes did differ significantly. In light of these findings, instructors should not ignore students’ school affect and burnout by paying attention only to their academic performance. Especially for the *moderate satisfaction/high frustration* and *low satisfaction/moderate frustration* profiles, intervention not only to enhance psychological needs satisfaction but also to alleviate need frustration is warranted. Since need satisfaction and need frustration can be considered independent experiences, this two-pronged approach may be most effective. Specific intervention approaches such as using noncontrolling language, providing choice, acknowledging one’s perspective and feelings, and assisting in setting an optimal level of challenge can be used (Teixeira et al. 2020).

### 4.4. Limitations and Future Directions

Although the present study has delivered some interesting findings, several limitations should be noted. First, our study used a cross-sectional design and collected data at a single measurement point; therefore, the stability of these profiles over time could not be examined. Future studies should use a longitudinal design (e.g., latent transition analysis) to further explore the remaining issues, such as whether and how the *moderate satisfaction/high frustration* and *low satisfaction/moderate frustration* profiles change over time. Second, we merged our interpretation of the need profiles without going into greater detail about the various types of needs. It is critical to understand that the satisfaction and frustration of each type of need may differ depending on the specific need profiles. In this way, dissecting the impacts of each type of need could aid in our understanding of the nuanced ways in which the three fundamental psychological needs appear. For instance, conducting qualitative interviews could offer a chance to better comprehend the unique needs of various students. Third, although we were able to identify gender and SES differences in the need profile membership, our data did not illuminate why such differences were found. Understanding how demographic characteristics may differentially shape profile membership would be beneficial for a targeted intervention. Thus, it is important for future research to explore the reasons that contribute to the observed difference. Fourth, participants were recruited from a single school and grade level. In this case, the Cronbach’s α coefficient was not very high for the autonomy satisfaction measure. Therefore, further investigation is required to determine the generalizability of these findings to students in other grades or school contexts. Future research should try to replicate these findings in more diverse student samples. Finally, the study was based solely on Chinese students, which limits its applicability to other student populations from diverse cultural backgrounds. As such, further investigations using more diverse groups are necessary to fully understand the implications of these findings.

## 5. Conclusions

The present study contributed to the existing SDT literature by identifying four distinct profiles of basic psychological needs satisfaction and frustration within the high school context. These profiles were further examined for their associations with school functioning and personal characteristics. The identified latent profiles in this study demonstrated clear patterns of need satisfaction and frustration, indicating that high school students can experience unique combinations of psychological needs. This study also added to the field by investigating differences in school functioning among the four latent profiles and highlighting that both low satisfaction and high frustration contribute to maladaptation. Furthermore, the findings revealed that students’ gender and socioeconomic status (SES) were significant predictors of profile membership. These findings can assist educators in gaining a better understanding of students’ patterns of psychological needs and enable the implementation of targeted interventions aimed at improving students’ enthusiasm and engagement in school. 

## Figures and Tables

**Table 1:** Descriptive Statistics, Reliabilities, and Correlation Coefficients of Main Variables.

	1	2	3	4	5	6	7	8	9	10
1. Autonomy satisfaction	--									
2. Autonomy frustration	−0.34 ***	--								
3. Relatedness satisfaction	0.51 ***	−0.30 ***	--							
4. Relatedness frustration	−0.31 ***	0.48 ***	−0.49 ***	--						
5. Competence satisfaction	0.49 ***	−0.31 ***	0.45 ***	−0.31 ***	--					
6. Competence frustration	−0.35 ***	0.52 ***	−0.27 ***	0.50 ***	−0.49 ***	--				
7. Positive affect	0.32 ***	−0.34 ***	0.24 ***	−0.24 ***	0.26 ***	−0.23 ***	--			
8. Negative affect	−0.29 ***	0.56 ***	−0.27 ***	0.46 ***	−0.33 ***	0.50 ***	−0.48 ***	--		
9. School burnout	−0.24 ***	0.51 ***	−0.20 ***	0.40 ***	−0.24 ***	0.43 ***	−0.28 ***	0.60 ***	--	
10. Achievement	0.02	−0.01	0.00	0.05	0.09 ***	−0.02	0.06 *	−0.10 ***	0.02	--
*α*	0.62	0.76	0.76	0.77	0.82	0.78	0.84	0.85	0.80	--
*M*	3.98	3.14	4.52	2.64	4.53	3.63	3.59	2.96	2.84	467.15
*SD*	0.85	0.91	0.90	0.98	0.86	1.04	1.15	1.05	1.01	81.60
Response range	1–6	1–6	1–6	1–6	1–6	1–6	1–6	1–6	1–6	136–654

*Note.* N = 1521 for all variables except achievement for which N = 1444. * p < 0.05, *** p < 0.001.

**Table 2:** The Model Fit Results of Latent Profile Analyses.

# of Profiles	LL	#par	AIC	BIC	ABIC	Entropy	VLMR	N Smallest Class
2	−11,320.57	19	22,679.14	22,780.35	22,720.00	0.74	<0.001	648
3	−11,091.83	26	22,235.66	22,374.17	22,291.57	0.72	<0.001	342
4	−11,000.33	33	22,066.67	22,242.46	22,137.63	0.75	0.45	72
5	−10,910.05	40	21,900.10	22,113.19	21,986.12	0.75	0.37	98
6	−10,844.70	47	21,783.39	22,033.76	21,884.46	0.78	0.47	54
7	−10,784.26	54	21,676.51	21,964.18	21,792.64	0.77	0.30	35
8	−10,741.63	61	21,605.27	21,930.22	21,736.44	0.76	0.59	33

*Note*. LL = model log likelihood; #par = number of free parameters; AIC = Akaike information criterion; BIC = Bayesian information criterion; ABIC = sample-size-adjusted BIC; VLMR = Vuong–Lo–Mendell–Rubin likelihood ratio test.

**Table 3:** Average Latent Profile Probabilities for Most Likely Profile Membership (Row) by Latent Profile (Column).

Profile	1	2	3	4	*N*
1 Low satisfaction/moderate frustration	0.848	0.000	0.116	0.036	316
2 High satisfaction/low frustration	0.000	0.878	0.122	0.000	356
3 Average all	0.060	0.059	0.858	0.023	777
4 Moderate satisfaction/high frustration	0.087	0.001	0.129	0.784	72

**Table 4:** Mean Values of Need Satisfaction and Frustration in Four Latent Profiles.

	Low Satisfaction/Moderate Frustration	High Satisfaction/Low Frustration	Average All	Moderate Satisfaction/High Frustration
Autonomy satisfaction	3.17 (1.00–6.00)	4.76 (2.75–6.00)	3.92 (2.00–5.75)	4.36 (2.25–6.00)
Relatedness satisfaction	3.52 (1.00–5.50)	5.26 (3.00–6.00)	4.59 (2.50–6.00)	4.58 (2.75–6.00)
Competence satisfaction	3.66 (1.00–5.75)	5.27 (3.50–6.00)	4.54 (2.50–6.00)	4.70 (3.00–6.00)
Autonomy frustration	3.67 (1.00–6.00)	2.27 (1.00–5.25)	3.23 (1.25–6.00)	4.07 (2.00–6.00)
Relatedness frustration	3.49 (1.00–6.00)	1.76 (1.00–4.25)	2.52 (1.00–4.50)	4.30 (3.50–6.00)
Competence frustration	4.37 (2.00–6.00)	2.57 (1.00–5.00)	3.73 (1.00–5.75)	4.44 (2.25–6.00)

*Note*. The response range is displayed within parentheses.

**Table 5:** Covariates Predicting Latent Profile Membership.

	Gender (1 = Female)				SES			
	Est.	OR	*SE*	*p*	Est.	OR	*SE*	*p*
Low satisfaction/moderate frustration vs. high satisfaction/low frustration	−0.211	0.810	0.219	0.385	0.059	1.061	0.024	0.013
								
Low satisfaction/moderate frustration vs. average all	−0.063	0.939	0.224	0.784	0.005	1.005	0.018	0.768
								
Low satisfaction/moderate frustration vs. moderate satisfaction/high frustration	−0.780	0.458	0.176	0.002	0.102	1.108	0.048	0.024
								
High satisfaction/low frustration vs. average all	0.148	1.159	0.249	0.522	−0.054	0.948	0.020	0.010
								
High satisfaction/low frustration vs. moderate satisfaction/high frustration	−0.569	0.566	0.208	0.037	0.044	1.045	0.039	0.257
								
Average all vs. moderate satisfaction/high frustration	−0.717	0.488	0.171	0.003	0.097	1.102	0.037	0.006

*Note.* Est. = logistic regression coefficient indicating the probability of being classified as a specific need profile versus the respective reference group, given specific categories/levels of the covariate. OR = odds ratio = e^(Est.)^.

**Table 6:** Means and Mean Differences in Outcome Variables among Profiles Based on Automatic BCH Approach.

	M_1_	M_2_	M_3_	M_4_	D (M_1_-M_2_)	D (M_1_-M_3_)	D (M_1_-M_4_)	D (M_2_-M_3_)	D (M_2_-M_4_)	D (M_3_-M_4_)
Positive affect	3.00	4.32	3.54	3.19	−1.33 ^***^	−0.54 ^***^	−0.19	0.78 ^***^	1.13 ^***^	0.35
Negative affect	3.64	2.02	2.98	4.11	1.63 ^***^	0.66 ^***^	−0.47	−0.96 ^***^	−2.10 ^***^	−1.13 ^***^
School burnout	3.35	2.06	2.88	3.82	1.29 ^***^	0.47 ^***^	−0.48	−0.82 ^***^	−1.77 ^***^	−0.94 ^***^
Achievement	460.37	466.19	469.02	478.86	−5.82	−8.65	−18.49	−2.83	−12.67	−9.84

*Note.* M_1_ = low satisfaction/moderate frustration, M_2_ = high satisfaction/low frustration, M_3_ = average all, M_4_ = moderate satisfaction/high frustration, D = difference between the means for the different profiles. ^***^ p < 0.001.

## Data Availability

Data will be made available on request.

## Appendix A. Items of Scales Used in the Present Study

**Autonomy satisfaction**
1. I feel a sense of choice and freedom in the things I undertake. 2. I feel that my decisions reflect what I really want.
3. I feel my choices express who I really am. 4. I feel I have been doing what really interests me.
**Autonomy frustration**
1. Most of the things I do feel like “I have to”.
2. I feel forced to do many things I wouldn’t choose to do.
3. I feel pressured to do too many things.
4. My daily activities feel like a chain of obligations.
**Relatedness satisfaction**
1. I feel that the people I care about also care about me.
2. I feel connected with people who care for me, and for whom I care.
3. I feel close and connected with other people who are important to me.
4. I experience a warm feeling with the people I spend time with.
**Relatedness frustration**
1. I feel excluded from the group I want to belong to.
2. I feel that people who are important to me are cold and distant towards me.
3. I have the impression that people I spend time with dislike me.
4. I feel the relationships I have are just superficial.
**Competence satisfaction**
1. I feel confident that I can do things well.
2. I feel capable at what I do.
3. I feel competent to achieve my goals.
4. I feel I can successfully complete difficult tasks.
**Competence frustration**
1. I have serious doubts about whether I can do things well.
2. I feel disappointed with many of my performances.
3. I feel insecure about my abilities.
4. I feel like a failure because of the mistakes I make.
**Positive school affect**
1. Most of the time, being in school puts me in a good mood.
2. I like being in school.
3. I am happier when I am at school than when I am not at school.
**Negative school affect**
1. I am often angry when I’m at school.
2. I often feel frustrated when I am doing school work.
3. School often makes me feel bad.
4. I often feel bored in school.
**School burnout**
1. I feel overwhelmed by my schoolwork.
2. I often sleep badly because of matters related to my schoolwork.
3. I brood over matters related to my schoolwork a lot during my free time.
4. The pressure of my schoolwork causes me problems in my close relationships with others.
